# Waist-to-Height Ratio as a predictor of cardiovascular and metabolic health in a pediatric population

**DOI:** 10.1371/journal.pone.0326772

**Published:** 2025-07-09

**Authors:** Per Morten Fredriksen, Asgeir Mamen, Nandu Goswami, Morten Lindberg

**Affiliations:** 1 University of Inland Norway, Faculty of Applied Ecology, Agricultural Sciences and Biotechnology, Rena, Norway; 2 Østfold University College, Faculty of Health, Welfare, and Organization, Halden, Norway; 3 Kristiania University College, School of Health Sciences, Oslo, Norway; 4 Gravitational Physiology and Medicine Research Unit, Division of Physiology and Pathophysiology, Otto Loewi Research Center, Medical University of Graz, Austria; 5 Center for Space and Aviation Medicine, College of Medicine, Mohammed Bin Rashid University of Medicine and Health Sciences, Dubai, United Arab Emirates; 6 Vestfold Hospital Trust, Central Laboratory, Tønsberg, Norway; University of Montenegro-Faculty of Medicine, MONTENEGRO

## Abstract

**Background:**

Understanding the relationship between central adiposity and health outcomes in children is crucial for early prevention of non-communicable diseases (NCDs). Waist-to-height ratio (WHtR) has emerged as a key anthropometric measure for predicting cardiovascular and metabolic health risks.

**Methods:**

The objective of this study was to investigate whether WHtR is associated with cardiovascular and metabolic risk markers, including HbA1c, CRP, lipid profiles, blood pressure, ferritin, and iron levels, in healthy children aged 6–12 years. The study further aimed to assess WHtR’s potential as a screening tool for identification of cardiometabolic risk.

**Results:**

An association between WHtR and unfavorable lipid profiles, with elevated total cholesterol and non-HDL cholesterol levels, alongside decreased HDL levels in the highest WHtR quartile, were displayed, suggesting increased cardiovascular risk. Similarly, the combination of high erythrocyte count, low hematocrit (HCT), high ferritin, and low iron, combined with high CRP may imply chronic inflammation due to adiposities. Elevated systolic and diastolic blood pressure values further underscore this cohort’s cardiovascular risks associated with central adiposity.

**Conclusion:**

WHtR’s ability to predict metabolic and cardiovascular risk factors highlights its potential as a simple, non-invasive screening tool in pediatric healthcare. Implementing WHtR in routine health assessments could provide an accessible and cost-effective method for early identification of at-risk children, enabling timely interventions to improve long-term health outcomes.

**Trial registration:**

Clinical Trial.gov. Identifier: NCT02495714. Registered 20 June 2015. https://register.clinicaltrials.gov/prs/app/action/LogoutUser?uid=U0002ORK&ts=13&sid=S0005MCN&cx=pc85td

## Introduction

Viewing illness on a continuum highlights the need for early detection and intervention. Although Body Mass Index (BMI) and Waist Circumference (WC) are commonly used to assess health risks, the Waist-to-Height Ratio (WHtR) is emerging as a more accurate measure of central adiposity and cardiometabolic risk. Unlike BMI, which overlooks fat distribution, WHtR is simple, effective, and independent of age and sex, making it a superior screening tool for identifying children at risk [1,2].

Blood tests and blood pressure measurements are vital for assessing cardiovascular and metabolic risks. Lipid profiles, glycated hemoglobin (HbA1c), and inflammatory markers may reveal metabolic health and the likelihood of non-communicable diseases (NCDs) such as diabetes and heart conditions. HbA1c is used to assess the risk of developing type 2 diabetes and other metabolic disorders. In pediatric populations HbA1c within the normal range have been associated with early metabolic dysregulation, particularly in the context of increasing adiposity and sedentary behavior [3]. Including HbA1c allows for evaluation of whether central adiposity correlates with impaired glycemic control in healthy children. This is relevant given the rising prevalence of childhood obesity. Understanding these associations may support the use of WHtR as a non-invasive predictor of metabolic risk [3]. Serum ferritin and iron levels are linked to systemic inflammation and oxidative stress, as high ferritin levels can indicate metabolic syndrome, insulin resistance, and increased risk of type 2 diabetes [4]. Additionally, inflammatory markers like C-reactive protein (CRP) are commonly elevated in individuals with obesity and are associated with an increased risk of cardiovascular disease [5].

Lipid profiles (total cholesterol, HDL, non-HDL) are crucial for evaluating atherosclerosis and cardiovascular disease risk [6]. Low HDL signals increased cardiovascular risk, while elevated non-HDL cholesterol correlates with plaque formation [7]. Systolic and diastolic blood pressure also matter as hypertension raises the likelihood of stroke, myocardial infarction, and other cardiovascular events [6,8]. Together, these indicators offer a broad view of cardiovascular and metabolic health, enabling early intervention in populations prone to NCDs. Monitoring them in children is particularly vital amid rising childhood obesity and metabolic syndrome [9], yet such assessments can be costly, time-consuming, and ethically challenging due to the invasiveness of blood sampling.

Using a reliable, non-invasive proxy for these risk factors could alleviate both ethical and logistical burdens of blood testing in children. It would also allow public health efforts to concentrate on high-risk groups, offering notable cost and efficiency benefits [10]. Anthropometric indicators, such as BMI, WC, WHtR, and body fat percentage, are widely used in public health due to their practicality, affordability, and proven predictive power for metabolic and cardiovascular risks [11]. WHtR is widely acknowledged for predicting NCDs in adults, yet its role in children remains less studied. Given the rising incidence of NCDs among younger populations, establishing WHtR’s predictive value in children is crucial for early detection and prevention. Its focus on central adiposity is especially relevant, as abdominal fat poses a higher health risk than total body fat [11].

The objective of this study was to investigate whether waist-to-height ratio (WHtR) is associated with key cardiovascular and metabolic risk markers in children aged 6–12 years. These markers included HbA1c, CRP, lipid profiles, systolic and diastolic blood pressure, ferritin, and iron levels. The study further aimed to evaluate WHtR’s potential as a simple, non-invasive screening tool for early identification of cardiometabolic risk in healthy pediatric populations.

## Materials and methods

The Health Oriented Pedagogical Project (HOPP), established in 2015, aimed to provide pupils in all elementary schools within Horten municipality, Norway, with 45 minutes of additional physical activity incorporated in the curriculum each school day, so called active learning. Detailed information regarding the recruitment process and intervention has been documented previously [12].

### Study sample

HOPP was initially planned as a longitudinal large-scale cohort study (2015–2021) with annual follow-up, targeting initially a total population of 2816 participants aged 6–12 at baseline. The total study sample comprised 2297 children (82%) after recruitment. Seven elementary schools in Horten municipality participated as the intervention group (n = 1545 at project start), receiving increased academically guided active learning. In comparison, two control schools from the greater Oslo area (n = 752 at project start) maintained normal activity. Data include 1–6 graders in 2015, 2–7 graders in 2016. From 2017 to 2020, the number of participants decreased annually due to students transitioning to secondary schools. As the COVID-19 pandemic ended data collection prematurely in 2020, this analysis utilizes data from January 15^th^, 2015, to May 15^th^, 2020. The variables included in the analyses serve as key predictors associated with the onset of metabolic diseases for children.

### Anthropometric measures

Body height was measured without shoes using a SECA 213 stadiometer (SECA GmbH, Hamburg, Germany). Body mass was measured barefoot, in light clothing, using a Tanita MC-980MA BIA electronic scale (Tanita Corporation, Tokyo, Japan). To compensate for the weight of the clothes, 0.4 kg was subtracted from the total weight. BMI was calculated based on body mass and height (kg/m^2^). Waist circumference (WC) was measured (cm) to the nearest 0.5 cm with an anthropometric non-elastic measuring tape after normal expiration at the level of the umbilicus (WHO method). WHtR was calculated by dividing WC (cm) by height (cm).

### Blood samples

Not all children or parents agreed to blood samples, hence, at baseline in 2015, n = 1344 were analyzed for all hematological parameters included in this study. In 2016, n = 1185 blood samples were taken. Between 2017 and 2020, blood samples were collected exclusively from 7th graders. Blood samples were collected in the non-fasting state between 8:00 a.m. and 1:30 p.m. without strenuous exercise before sample collection. Phlebotomists drew blood from the antecubital vein in 4 mL K2EDTA tubes (Vacuette®, Greiner Bio-One, Austria). Samples were collected in the respective schools and analyzed with standard procedures at the central laboratory at Vestfold Hospital Trust (accredited by NS-EN ISO 15189). All analytes were individually accredited and participated in external proficiency testing schemes from NOKLUS [13]. The hematology analyses were performed on Sysmex XE 2100 (Sysmex Corporation, Kobe, Japan) with reagents from the supplier.

### Blood pressure

An automatic blood pressure monitor (Model M6 Comfort IT, Intellisense HEM-7322U-E; Omron Healthcare Co. Ltd., Kyoto, Japan) equipped with an Intelli Wrap Cuff (HEM-FL31, Omron Healthcare Co. Ltd.) was used for the measurements. The cuff was initially attached to the left upper arm, and BP was measured up to three times if pressure detection failed or the results were outside the normal age-adjusted ranges.

### Analyses

Preliminary descriptive analyses were conducted to assess the stability of WHtR across the study period. These analyses indicated minimal clinically relevant variation in WHtR, supporting its use as a consistent measure across age and time in subsequent regression models. As the study focused on promoting physical activity and academic performance rather than altering WHtR, all collected data were included in the analyses. Moreover, attempting to reduce weight or WC in healthy, growing children would be ethically unjustifiable. Due to the study design, repeated measures were treated as independent observations in regression models to assess secular trends rather than individual longitudinal changes. This approach enhances interpretability and statistical power but does not adjust for within-subject correlation.

Shapiro-Wilk normality test was used in all continuous variables. Pearson correlation revealed low correlation except between total cholesterol and non-HDL (r = 0.85). As such, total serum cholesterol was chosen in the analyses. The health-related outcomes of WHtR quartiles using ANOVA post-hoc tests to determine statistically significant differences between WHtR-quartiles was used. Visual inspections using error bars with a 95% confidence interval (CI) were also presented. Statistical analysis was performed using SPSS version 28 (IBM, Armonk, NY, USA), with a significance level of α ≤ 0.05.

Average WHtR, age, sex, and socioeconomic status (SES) were employed as independent variables in regression models, while the health outcomes of HbA1c, cobalamin (B12), holotranscobalamin (B12A), erythrocytes (Ery), hemoglobin (Hb), total serum cholesterol (cholesterol), high density lipoprotein (HDL), hematocrit (HCT), C-Reactive Protein (CRP), systolic blood pressure (BPsys) and diastolic blood pressure (BPdia) were used. Values of beta (β), alpha (α) and Cohen’s *f*^*2*^ were reported to indicate the slope, statistical significance, and the effect size of the relationships.

The original WHtR data had limited variability reducing the predictive power of the regression model and leading to exaggerated effect sizes. Small variations were amplified, complicating interpretation. To address this, WHtR was rescaled (multiplied by 100) to enhance interpretability, ensuring biologically meaningful and statistically reliable associations [14]. This adjustment improved the model’s robustness and practical applicability.

### Ethics and consent

The study follows the ethical guidelines of the Declaration of Helsinki and was approved by the Regional Committee for Medical Research Ethics (REK, ref. no. 2014/2064). It is registered in ClinicalTrials.gov (NCT02495714) since June 20, 2015. Identifiable data were replaced with unique codes, securely stored, and deleted before analysis. Written informed consent was obtained from all participants’ legal guardians.

## Results

### Sample

Across 6 years, and based on the baseline study sample of 2297 children, a total of 8421 measurements were included in the descriptive secular analyses. Of these, 7075 (84%) measurements had a WHtR below 0.5, and 1346 (16%) above 0.5. These findings align with national reference values for overweight prevalence in children using isoBMI [15]. The mean WHtR across all measurements was 0.45 (SD: 0.04, range: 0.26–0.74), with 16% of the total observations exceeding the established 0.5 cutoff for increased cardiometabolic risk (Table 1). This aligns with national reference values for childhood overweight prevalence. Notably, mean weight and height increased across study years, reflecting normal growth trends. The relatively narrow WHtR distribution highlights the need for rescaling to enhance interpretability in statistical models. These findings reinforce WHtR as a practical measure for assessing central adiposity in pediatric populations.

**Table 1:** Descriptive statistics for weight, height, and weight-to-height ratio (WHtR) from 2015 to 2020.

		N	Mean	SD	N	Mean	SD	N	Mean	SD	N	Mean	SD	N	Mean	SD	N	Mean	SD
		2015			2016			2017			2018			2019			2020		
**Boys**	**Weight (kg)**	1150	33.4	9.22	1075	38.4	10.56	823	39.1	10.28	609	41.2	10.28	445	43.3	10.17	282	45.8	10.58
	**Height (cm)**	1150	138.7	11.49	1085	144.5	11.64	837	146.4	10.38	613	149.0	9.36	445	152.4	9.18	281	154.8	8.52
	**WHtR**	1099	0.45	0.04	1071	0.45	0.05	829	0.45	0.05	592	0.46	0.05	434	0.45	0.05	271	0.45	0.05
**Girls**	**Weight (kg)**	1121	33.3	9.90	1041	38.2	11.00	785	39.2	10.58	586	41.4	10.72	401	43.6	9.82	261	46.6	10.56
	**Height (cm)**	1122	138.3	12.05	1049	144.0	12.17	810	146.2	10.71	590	149.0	9.74	402	152.3	8.53	258	154.9	8.17
	**WHtR**	1080	0.45	0.05	1042	0.44	0.05	795	0.45	0.05	571	0.45	0.05	388	0.45	0.05	247	0.44	0.06

Waist-to-height ratio (WHtR), Standard Deviation (SD), N = number of measurements.

### Quartiles

**Fig 1:**
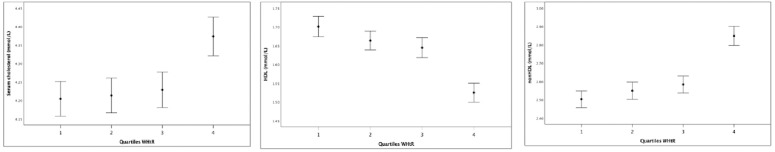
Quartiles of Waist-to Height Ratio (WHtR) compared to total serum cholesterol (left), high density lipoprotein (HDL, middle) and total serum cholesterol minus HDL (non-HDL, right).

**Fig 2:**
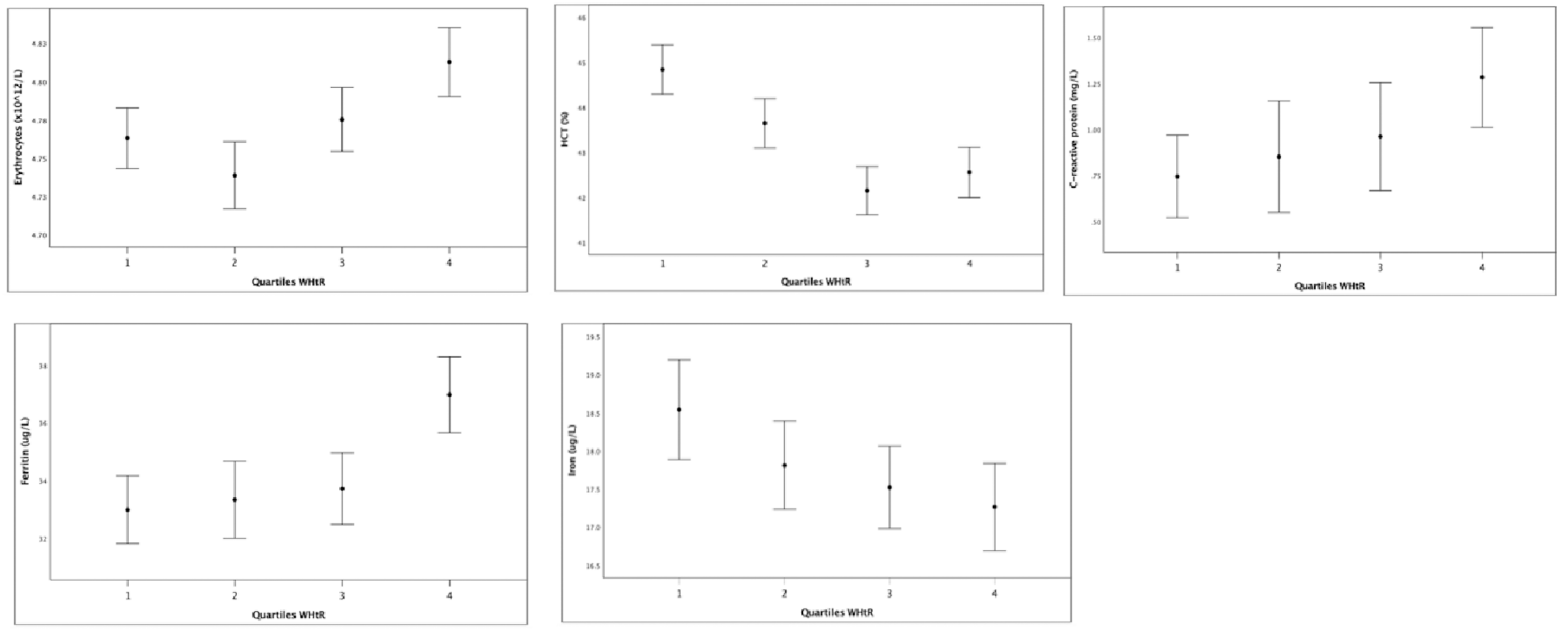
Quartiles of Waist-to-Height Ratio (WHtR) compared to erythrocytes (ERY, top left), hematocrit (HCT), top middle), C-reactive protein (CRP, top right), ferritin (bottom left) and iron (bottom right).

**Fig 3:**
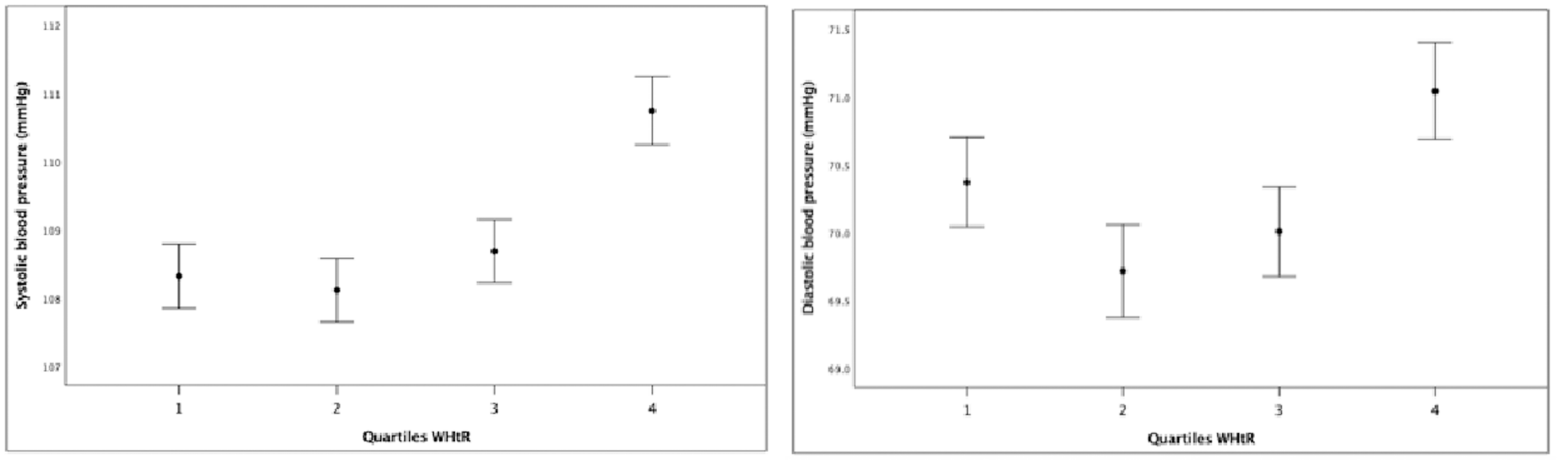
Quartiles of Waist-to-Height Ratio (WHtR) compared to systolic (left) and diastolic (right) blood pressure. Both variables clearly show an increase with higher WHtR.

Quartile four (Q4) of WHtR showed significant effects on all cholesterol variables, with higher total serum cholesterol (p < 0.001), higher non-HDL (p < 0.001), and lower HDL (p < 0.001), indicating an unfavorable cholesterol profile in children (Fig 1). Additionally, high erythrocyte count (ERY; p < 0.001), low hematocrit (HCT; p < 0.001), elevated ferritin levels (p < 0.001), and reduced serum iron (p = 0.023), along with increased CRP levels (p = 0.042), were observed (Fig 2). Blood pressure analysis revealed significant increases in both systolic (p < 0.001) and diastolic (p < 0.001) measures with increasing WHtR (Fig 3). The values show a negative impact of high values of WHtR, represented by Q4, on all three variables.

### Regression models

The regression models analyzed the effects of sex, age, SES, and WHtR on various blood biomarkers in healthy children aged 6–12 years. These models provide insights into highlighting the role of WHtR as a predictor of cardiometabolic risk. The regression model showed no significant effects of sex, SES, or WHtR on HbA1c levels. Also, age displayed a significant effect of age, however, the lack of clinically meaningful changes suggests that HbA1c is stable across age in this cohort. Vitamin B12 levels are higher in younger children and those from higher SES backgrounds. WHtR is negatively associated with B12, suggesting potential nutritional concerns in children with higher central adiposity. Higher parental SES correlates with lower WHtR, possibly reflecting better nutrition and B12 status. No significant effect of sex was found for B12. For active B12, boys and younger children displayed higher levels, though effect sizes were small. These findings imply the importance of addressing nutritional quality in children with higher WHtR.

### Oxygen transport

Erythrocyte count (Table 2) increases with age and is lower in girls, though with limited clinical relevance. WHtR shows a minor negative association but lacks significant clinical impact. These findings likely reflect age-related changes in erythrocyte production with slight modulation by body composition. SES had a significant positive effect on HbA1c, B12 and B12A. Especially B12 and B12A showed a clinical important effect, perhaps indicating better nutrition standards with increasing parental SES. HCT levels (Table 4) was positively associated with age and lower in girls, with no significant WHtR association. SES, however, indicate an increase in HCT with improved parental SES.

**Table 2:** The table display beta, alpha and Cohen’s *f*^2^ for the effect of sex, age, socioeconomic status, and waist-to-height ratio (WHtR) on the blood samples of HbA1c, B12, B12A and Ery.

	HbA1c			B12			B12A			Ery		
	β	α	*f* ^2^	β	α	*f* ^2^	β	α	*f* ^2^	β	α	*f* ^*2*^
**Sex**	−0.01	0.250	−0.03	−4.08	0.698	−0.01	−10.3	<0.001	−0.10	−0.03	0.006	−0.06
**Age**	−0.01	0.017	−0.05	−31.1	<0.001	−0.31	−5.48	<0.001	−0.20	0.022	<0.001	0.15
**SES**	0.02	0.010	0.06	13.99	0.028	0.07	7.17	<0.001	0.12	−0.01	0.126	−0.03
**WHtR**_**100**_	0.01	0.279	0.03	−2.79	0.019	−0.07	−0.29	0.296	−0.03	0.01	<0.001	0.09

Socioeconomic status (SES), Waist-to-height ratio (WHtR), Glycated hemoglobin (HbA1c), Cobalamin (B12), Holotranscobalamin (B12A), Erythrocytes (Ery), Beta-value (β), p-value (α), Effect size (Cohen’s *f*^*2*^).

**Table 4:** The table display beta, alpha and Cohen’s *f*^*2*^ for the effect of sex, age, socioeconomic status, and waist-to-height ratio on the blood samples of high-density lipoprotein, non-HDL, hematocrit, and CRP.

	HDL			Non-HDL			HCT			CRP		
	β	α	*f* ^*2*^	β	α	*f* ^*2*^	β	α	*f* ^*2*^	β	α	*f* ^*2*^
**Sex**	−0.11	<0.001	−0.16	0.19	<0.001	0.15	−0.90	0.001	−0.07	−0.15	0.350	−0.03
**Age**	−0.01	0.002	−0.07	−0.02	0.753	−0.07	1.77	<0.001	0.52	−0.10	0.023	−0.06
**SES**	−0.06	0.499	−0.02	−0.03	0.131	−0.03	0.74	<0.001	0.10	0.03	0.756	0.01
**WHtR**_**100**_	−0.02	<0.001	−0.21	0.03	<0.001	0.21	−0.05	0.082	−0.04	0.05	0.006	0.07

Socioeconomic status (SES), Waist-to-height ratio (WHtR), High density lipoprotein (HDL), Hematocrit (HCT), C-Reactive Protein (CRP). By subtracting HDL from total serum cholesterol, non-HDL may be used as a substitute for low-density lipoprotein (LDL), Beta-value (β), p-value (α), Effect size (Cohen’s *f*^*2*^).

### Iron

Ferritin (Table 3) is positively associated with WHtR, suggesting a potential inflammatory response in children with higher WHtR. Age also predicts ferritin, though with minimal clinical impact, while sex showed no significant effect. Ferritin did display an increase with higher parental SES, possibly reflecting better nutrition status. For iron (Table 3), WHtR has a slight significant negative association, possibly linked to subclinical inflammation or diet with increased abdominal fat. Girls had lower iron levels. No significant effects of age or SES were revealed for iron. Hb levels (Table 3) showed no associations with WHtR, sex or SES, though age had a significant positive effect, consistent with developmental trends.

**Table 3:** The table display beta, alpha and Cohen’s *f*^*2*^ for the effect of sex, age, socioeconomic status, and waist-to-height ratio on the blood samples of ferritin, iron, Hb, and serum cholesterol.

	Ferritin			Iron			Hb			Cholesterol		
	β	α	*f* ^*2*^	β	α	*f* ^*2*^	β	α	*f* ^*2*^	β	α	*f* ^*2*^
**Sex**	−0.58	0.45	−0.02	0.69	0.048	0.06	−0.04	0.238	−0.03	0.08	0.008	0.06
**Age**	0.59	0.03	0.07	0.12	0.229	0.04	0.11	<0.001	0.298	−0.02	0.049	−0.04
**SES**	1.21	0.01	0.06	0.16	0.450	0.02	−0.01	0.906	−0.01	−0.03	0.070	−0.04
**WHtR**_**100**_	0.40	<0.001	0.11	−0.11	0.007	−0.08	0.003	0.357	0.020	0.01	<0.001	0.09

Socioeconomic status (SES), Waist-to-height ratio (WHtR), Hemoglobin (Hb), Total serum cholesterol (cholesterol), Beta-value (β), p-value (α), Effect size (Cohen’s *f*^*2*^).

### Lipids and inflammation

WHtR was positively associated with total serum cholesterol (Table 3), suggesting a role in lipid dysregulation, though with minimal clinical impact. Boys had higher cholesterol levels, while age was borderline significant, SES was borderline non-significant. HDL (Table 4) was influenced by sex and age, with girls having lower levels and both sexes showing a decline with age, though with limited clinical relevance. HDL also had a negative association with WHtR, linking central adiposity to lower protective lipid profiles. Non-HDL (Table 4) showed no effect of age or SES but was significantly higher in girls. WHtR was positively associated with non-HDL, reinforcing its connection to adverse lipid profiles, albeit with a small effect.

CRP levels (Table 4) are positively associated with WHtR, suggesting an inflammatory response in children with higher central adiposity. Age also showed significant effect, with lower values for girls. No effect was found for sex or SES.

### Blood Pressure

Age was significantly associated with both systolic and diastolic blood pressure, with older children showing higher values (Table 5). Girls had lower systolic but no difference in diastolic pressure. WHtR was positively associated with both systolic and diastolic blood pressure (Table 5), highlighting the impact of central adiposity on vascular health. SES had borderline non-significant effect on systolic blood pressure, however, did show positive effect on diastolic pressure.

**Table 5:** The table display beta, alpha and Cohen’s *f*^*2*^ for the effect of sex, age, socioeconomic status, and waist-to-height ratio on systolic and diastolic blood pressure.

	BPsys			BPdia		
	β	α	*f* ^*2*^	β	α	*f* ^*2*^
**Sex**	−1.32	<0.001	−0.06	0.37	0.080	0.02
**Age**	1.56	<0.001	0.25	0.57	<0.001	0.13
**SES**	−0.29	0.092	−0.02	−0.34	0.008	−0.04
**WHtR**_**100**_	0.23	<0.001	0.10	0.08	<0.001	0.049

Socioeconomic status (SES), Waist-to-height ratio (WHtR), Systolic blood pressure (BPsys) & Diastolic blood pressure (BPdia), Beta-value (β), p-value (α), Effect size (Cohen’s *f*^*2*^).

## Discussion

WHtR was negatively associated with protective lipid profiles, showing lower HDL and higher total and non-HDL cholesterol. It also correlated with elevated CRP, indicating inflammation, and higher blood pressure, reflecting central adiposity’s vascular impact. Minor but significant associations with ferritin and iron suggest possible subclinical inflammation or dietary factors. Age influenced most outcomes, and sex differences were observed in lipid profiles and blood pressure. These findings support WHtR as a useful tool for identifying at-risk children while highlighting the need to account for age and sex in interpretation. Higher SES also displayed positive effects on oxygen transport variables, and ferritin but not iron.

### Sex differences

Sex differences in cardiometabolic markers were observed, with girls showing lower erythrocyte counts and Hb levels, likely due to hormonal influences on erythropoiesis. These differences, consistent with previous findings, generally remain within normal physiological ranges and have minimal clinical impact [16]. Elevated total cholesterol is a known risk factor for cardiovascular disease, with childhood levels potentially predicting future risk [17]. Boys showed higher total cholesterol and HDL, but the small effect size suggests limited clinical relevance. Non-HDL cholesterol, encompassing all atherogenic lipoproteins, is a stronger marker of cardiovascular risk than LDL alone [7]. Girls had higher non-HDL levels, aligning with previous findings, suggesting a less favorable lipid profile. While girls typically show higher total cholesterol and non-HDL, they also tend to have higher HDL, which may counterbalance the risk [18].

Sex had no significant effect on CRP or iron, though research links both sex and obesity to elevated CRP [19]. Limited studies on sex differences in CRP among healthy children make conclusions difficult. Ferritin showed a borderline non-significant effect (p = 0.051) with a high beta value. Boys had higher systolic blood pressure, while diastolic pressure showed no sex differences. High blood pressure prevalence is reported at 10.2% in boys and 7.6% in girls. Some studies suggest girls may have higher diastolic pressure, highlighting variability in sex differences across populations [20].

### Age differences

Age significantly influenced multiple outcomes, reflecting expected physiological changes in childhood. Older children had higher erythrocyte counts, Hb levels, and blood pressure, aligning with growth patterns [19]. Age had no effect on serum iron but was positively associated with ferritin, though with minimal impact. Some suggest ferritin changes are more influenced by diet, inflammation, or socioeconomic factors than age alone [21]. Low-grade inflammation may also confound ferritin levels, particularly in populations with obesity or subclinical infections [22]. HDL declined with age, suggesting early unfavorable lipid changes, while non-HDL cholesterol and CRP remained unaffected.

### SES differences

SES had a limited impact on cardiometabolic outcomes but was positively associated with B12 and B12A, indicating a link between higher parental SES and better nutrition. However, SES showed no significant effects on cholesterol, blood pressure, or inflammatory markers like CRP. The absence of SES influence on lipid profiles aligns with findings in other healthy cohorts [23]. While SES appears to have minimal effects on cardiometabolic risk, its role in shaping dietary habits and nutrition warrants further study.

### WHtR

WHtR often outperforms BMI in predictive models by accounting for fat distribution, a key factor overlooked by BMI [10]. Also, BMI requires age- and sex-specific adjustments, whereas WHtR uses a standard cutoff of 0.5 that is broadly accepted [24]. Despite this, the validity of WHtR across ethnic groups remains debated: Asian populations may be at risk with lower WHtR, while African and Hispanic groups may need higher cutoffs. Relying on a single threshold may therefore fail to capture risk in all populations, calling for more ethnicity-specific research. Still, pediatric studies indicate that WHtR is a stronger predictor of fitness level than WC or BMI [25].

In healthy cohorts with a narrow WHtR range (0.3–0.7), using a one-unit increment in regression may inflate effect sizes. Rescaling WHtR by multiplying it by 100 improved interpretabilities by converting small variations into more meaningful units, reducing exaggerated effect sizes, and ensuring biologically plausible associations in the regression models. This conversion is supported in the field of statistics [14].

Higher WHtR is linked to metabolic changes associated with adiposity. Visceral fat secretes pro-inflammatory cytokines (IL-6, TNF-α), promoting systemic inflammation and insulin resistance, which can lower HDL and raise non-HDL cholesterol, increasing cardiovascular risk [26]. This align with the elevated CRP findings that suggests chronic low-grade inflammation linked to excess adiposity [9]. This study confirmed WHtR’s associations with adverse lipid profiles, reinforcing its role in early risk detection [27].

WHtR-related systemic inflammation and cytokine elevation may impair erythropoiesis by suppressing erythropoietin and disrupting iron metabolism, reducing reticulocyte production [22]. Increased erythrocyte count could reflect a compensatory response to low-grade hypoxia, common in individuals with excess adiposity [28]. This dysregulation in red blood cell turnover and iron homeostasis may indicate early metabolic dysfunction [29]. The study’s link between higher WHtR and elevated ferritin aligns with research associating increased ferritin with body iron stores, chronic inflammation, and metabolic syndrome [30].

The reduction in serum iron despite elevated ferritin suggests inflammation-driven iron dysregulation. Adipose tissue increases hepcidin production, reducing iron absorption and trapping iron in storage, raising ferritin while lowering circulating iron [31]. The body may also sequester iron as a defense against pathogens, decreasing its availability even when ferritin is high [32]. Additionally, rapid growth in childhood increases iron demand for blood volume and muscle mass expansion [33]. Children with high adiposity may still face iron deficiency if intake is insufficient.

Also, high WHtR is linked to increased hypertension risk in adulthood, a major predictor of cardiovascular disease [8]. While most healthy children maintain normal BP, those in WHtR Q4 in the present study had higher systolic and diastolic BP values, suggesting a potential hypertension risk [8]. This suggest that early monitoring of BP in children in the upper quartile may have preventive effects on cardiovascular health [34]. Monitoring BP alongside WHtR allows for timely interventions, such as dietary adjustments, increased physical activity, and weight management, to reduce future cardiovascular risks [35].

In sum, WHtR shows potential as an early marker for chronic disease risk in children due to its links with unfavorable values of lipoproteins, inflammation, iron metabolism, blood pressure, and erythropoiesis. As both a risk factor and proxy for other risks, WHtR provides a simple, cost-effective method for assessing cardiometabolic health in children. Given the variability in pediatric metabolic syndrome definitions, WHtR could offer a standardized screening measure across diverse populations.

### Limitations

This study offers valuable insights into WHtR and cardiometabolic risk in children but has some limitations. While capturing multi-year trends, it does not track individual longitudinal changes, limiting insight into the effects of growth and puberty. Additionally, the absence of dietary data prevents assessment of nutritional influences on body composition and metabolism. Genetic predisposition to obesity and metabolic disorders is also unaccounted for, making it difficult to separate hereditary from environmental factors. Furthermore, blood biomarkers were collected in a non-fasting state, which may have affected lipid levels, though not HbA1c. Selection bias is also possible as not all children participated in blood sampling. Despite these limitations, the study’s large sample size, extended data collection, and comprehensive assessments strengthen its reliability.

## Conclusion

This study highlights WHtR as a valuable marker for identifying cardiometabolic risk in children. The narrow WHtR range in this healthy cohort required rescaling to improve interpretability, reinforcing its predictive reliability. Higher WHtR was linked to lower HDL, higher total and non-HDL cholesterol, increased CRP, and elevated blood pressure, indicating early vascular and inflammatory changes. While sex and age differences were observed, their clinical impact was minor. These findings support integrating WHtR into routine pediatric assessments for early intervention, while further research should explore dietary and genetic influences on these associations.
